# An overview of Indian research in bipolar mood disorder

**DOI:** 10.4103/0019-5545.69230

**Published:** 2010-01

**Authors:** Prasad G. Rao

**Affiliations:** Department of Psychiatry, Schizophrenia and Psychopharmacology Division, Asha Hospital, Banjara Hills, Hyderabad, India

**Keywords:** Bipolar mood disorder, manic depressive psychosis, genetic studies, clinical picture and course, pharmacological evidence

## Abstract

This review has been done after careful research of articles published in indian journal of psychiatry with the search words of manic depressive psychosis and bipolar mood disorder. Many articles in the following areas are included: 1) Etiology: genetic studies: 2) Etiology – neuro psychological impairment: 3) Adult bipolar disorder 4) Epidemological 5) Clinical picture – phenomenology: 6) Course of bipolar mood disorder: 7) Juvenile onset bipolar affective disorder 8) Secondary mania: 9) Clinical variables and mood disorders: 10) Disability: 11) Comorbidity: 12) Treatment: biological 13) Recent evidence: 14) Pharmacological evidence in special population. Though there seems to be significant contribution, there are still lot of areas which need careful intervention. The findings in various studies from the indian point of view are reviewed.

## INTRODUCTION

In 1896, Kraeplin reported ‘manic-depressive psychoses’ as a circumscribed disease entity. Ever since, manic depressive psychosis, or the current term used nosologically as ‘bipolar’ mood disorder, has been studied in the Indian perspective. Though it seems like there is no orderliness in the research pursuit of understanding this disorder in the Indian context, one gets an impression that all aspects like nosology, clinical syndromes, course, pharmacological and in special populations as well, were attempted to be looked at from the Indian context. This review is an attempted peep into Indian context research.

A review of the studies on bipolar mood disorders (Manic Depressive Psychosis) over the last many decades, published in Indian Journal of Psychiatry, conveys the feeling that over the years, though many aspects have been studied, there is no consistency in reports across the country. Reviewing research would find case reports to studies and reviews. Representative studies have come from across the country and in a way present the diversity of various centers; it doesn’t seem to come from one center alone. We attempt to collect all the studies published in this area by going through as many old issues of Indian Journal of Psychiatry as were available.

## ETIOLOGY: GENETIC STUDIES

Dermatoglyphics as a diagnostic tool studied 100 normal, 60 manic depressive psychosis subjects, 30 unipolar subjects, 30 bipolar subjects at Chandigarh.[1] Details of socio demographic, family h/o, and diagnosis based on ICD 8 1965 and DSM II 1968 were used. Finger and palm prints were studied as per standard guidelines. Interesting findings emerged. There were differences between unipolar and bipolar groups *vis a vis* normal groups in various measures. The significance found was - Patients with manic depressive psychosis and with positive family h/o had differed as a group with negative family h/o, making the authors to conclude that genetic factors do play a role in manic depressive psychosis.

### Lithium response as[2] a prophylactic agent in a 25-patient cohort

Among 25 patients with 1.4 years of optimum serum level of 0.6 M.EQ/L, precise and absence of positive h/o of psychiatric illness in first degree relatives of family members was seen. The patients were divided into two groups based on chart reviews in responders and non responders. Responders significantly had positive family h/o affective illness. Though this study had only a small sample, for the first time in Indian literature, it commented on the genetic aspects of pharmacological response.

Genomic imprinting in bipolar affective disorder was studied by R. Kumar *et al*. 2000 at Central Institute of Psychiatry (CIP), Ranchi;[3] in the first episode, bipolar affective disorder was diagnosed according to DSM IV 1994 criteria and without any comorbidity. This is the first study in bipolar mood disorder in genomics. Out of 79 conservative cases with first degree, the results of this study did not establish a phenomenon of imprinting in non mendelian patterns of inheritance leading the authors to conclude that bipolar disorder is heterogeneous. A very small sample size might have made it difficult to comment on the results.

### Etiology-neuro psychological impairment

M Taj and Padmavathi R[4] have assessed neuro psychological-neuro cognitive impairment in 30 patients of bipolar disorder and 30 matched controls for age, gender, education with no past, present or family history of psychiatric illness. This study reports that patients with bipolar disorder in remission and maintenance phases, with mood stabilizers, have impaired attention memory and executive functions. The authors conclude that cognitive dysfunctions contribute to social and occupational difficulties on one hand and reduced insight and increased non adherence and risk of relapse. The contribution of the drugs in the cognitive dysfunction could not be commented on. Psychoeducation and cognitive rehabilitation was suggested for increasing adherence to drugs-mood stabilizers for prevention of relapse.

### Adult bipolar disorder

#### Epidemological

Chopra HD *et al*.[5] have attempted to study the socio economic status and manic depressive psychosis, in Ranchi in a private psychiatric hospital setting, the out patients and in patients using Kuppuswamy scale for urban and Pareek and Trivedi scale for rural study and the largest per cent of patients fit into socio class III. In this study 100 patients were studied. Interestingly, this is one study which looked at the relation of socio economic classes to any psychiatric disorder and concluded that there is a higher representative in middle class as regards to manic depressive psychosis at that time.

### Clinical picture-phenomenology

Chatterjee and Kulhara[6] have reported sympto matology and symptom resolution in short term (90 days). In this prospective study, present state examination schedule and Bach Rafaelson Mani scales were used in 40 patients diagnosed by DSM III criteria for manic episode. All patients were unitedly managed with chloropromazines equivalence ranging from 300 to 900 mgs.

In this study, the behavior affect, speech, delusions and hallucinations was done using PSE Items and the periodic assessments helped to show the resolution time of each symptoms preponderance of male subjects was noted. Mean duration of current episode was shorter in males compared to females.

Symptomatologically, Indian patients differed significantly as having distractibility as symptoms and more of embarrassing behavior. Hostile irritability is the dominant affect, 62.5% had one or more delusions. In this sample, which is more than many studies reported by other international studies notably Taylor and Abrams (1973) Carlson and Goodwin (1973) in terms of recovery by four weeks, delusions and hallucinations disappear.

Authors comment that where some symptoms resolving quickly, where some resolve quickly, others much slowly. Only 15% remained hospitalized for 90 days, and majority got discharged early; authors conclude that in India Mania patients resolve early with treatment. This is one study which systematically studied the clinical symptomatology in the Indian context, at PGIMER Chandigarh.

R Kumar *et al*.[7] have carried out a systematically studied the phenomenology of mania and used a factor analysis approach to derive the clusters and arrived at three cluster groups using ICD-10 criteria; and 100 consequent patients diagnosed with bipolar disorder or manic episode were the study subjects. Predominantly male (77%), all patients were rated on the scale of manic states (Cassidy *et al*. 1998).

There were three factors which had significant variance-factor number one had motor activity, pressured speech, racing thoughts, increased sexuality, increased contact as the clinical symptoms and, in essence, picks up psychomotor acceleration as the main factor and has largest variance in the patient sample. The second factor picked up thought disorder and psychosis with grandiosity, lack of insight and paranoid factors. The third factor, which has about 13.8% variance, represented mood with large percent having irritability (82%), euphoria (51%), aggression (70%), anxiety (59%) as phenomenology, the study has a good sample size, good methodology representing Indian population.

### Course of bipolar mood disorder

R Kumar *et al*.[8] have studied, in a 28-day study from day 0 of index diagnosis of bipolar mania diagnosed according to DSM IV, and attempted to study gender differences in the resolution of mania. Rao TSS, Rao V, Shivamoorthy S, Kuruvilla K (1993), carried out a genetic study using pedigree method in patient diagnosed to behaving bipolar affective disorder and evaluated 76 individuals. They opined that the gene borne influence was discernable in their analysis.[9] In contrast to the Chatterjee and Kulhara study[6] the methodology used in this study was by using scale of manic states by Cassody *et al*. and using survival plot of resolution. The authors attempted to see gender differences in resolution of manic symptoms, in sample of 40 (24 males and 16 females). The attempt included to see the differences both in severity and symptomatology across the genders. There were significant difference at Index rating on certain items in females-viz. increased sexuality and aggression.

Srinivasan *et al*.[10] have attempted to identify the differences of phenomenology, family history between unipolar mania and bipolar mania. They did not find any differences regards to various phenomenologies between two diagnostic groups and concluded that from their results they are homogenous. R Kumar and Dayaram[7] studied the evolution of manic symptoms in the first time diagnosed fresh episode of mania in bipolar affective disorders, defining the evolution in a sample of 98 patients (81 males). No consistent pattern of evolution was identifiable in this study. Median duration of evolution was 45 days, however, females and patients with significant life events had a shorter evolution period.

A naturalistic course of bipolar disorder in rural India[11] was reported by Chopra M P *et al*. This data is from the Primary Health Centre, Sakalwara, adapted at NIMHANS, 27 patients of 34 patients evaluated had not received any treatment at all, though there were many episodes 15% of patient has had rapid cycling, episodes of manic accounted for 72% of episodes. None of the variables examined could predict the total number of episodes. However, patients receiving psycho pharmacological agents are likely to develop rapid cycling. A mania predominant course was observed in this study cohort.

### Juvenile onset bipolar affective disorder

There are very few studies in this area, published in Indian journal of Psychiatry, except for case reports.

Narasimha Rao IV L *et al*.[12] have for the first time reported three case reports. They concluded that phenomenologically the three cases were similar to adult manic depressive psychosis and also lithium is likely to give good response just in adult manic depressive psychosis. Vijaysagar K J[13] has reported a case of juvenile onset bipolar affective disorder. It is pertinent to mention that there were reports of course and phenomenology published in other journals from NIMHANS and can be considered as the data from India, from National Institute of Mental Health and Neuro Sciences, Bangalore. This data suggests the occurrence of discreet, short lived episodes of mania, a high rate of recovery defined and low rate of chronicity. Interestingly, the above findings are in distinct contrast with the findings of western studies. Likewise, Indian studies report low incidence of rapid cycling, less psychosis and less of mixed symptoms as compared to the western studies.

### Secondary mania

Tricyclic anti depressant-induced mania was presented in a case report of four mono polar depressed patients who developed mania after tricyclic anti depressant therapy.[14] This study was the first to report the anti depressant induced mania.

There are many case reports of mania occurring due to other medical conditions or due to drugs were reported-Chronic mania due to polio encephalomyelitis,[15] Tertiary Syphilis.[16] Venilafaxine-induced mania,[17] anti depressant-induced mania,[18] Bupropion,[19] M Arora *et al*.[20] have tried to show, citing two cases, that significant life events can precipitate manic episodes.

Yadav R and Pinto C[21] report a case of treatment emergent dyskinesia in a patient with mania occurring in Parkinson’s disease. In this case there were two episodes of mania in a seven-year gap; first episodes occurring at the start of the treatment and in the second episode there were issues of dyskinesia due to Parkinson’s disease treatment.

Mania in HIV infection was reported by Venugopal D *et al*.[22] where the critical issues of the management and diagnosis were discussed. Chopra V K *et al*.[23] discussed bipolar disorder associated with tuberous sclerosis in a seven-year-old, as a secondary mania etipathogenous. There are also a few case reports of secondary mania following encephalitis,[24] seen following stroke,[25] Turners syndrome.[26]

Mania starting during hypno therapy[27] into mania was reported in a single case report.

### Clinical variables and mood disorders

Gurmeet Singh *et al*.[28] have attempted to see the relation of A, B, O blood groups with bipolar and unipolar affective disorders, in 200 consecutive patients. They found significantly increased frequency of blood group O in manic depressive psychosis and lesser frequency of blood group A in comparison to normal controls and unipolar group. The authors conclude that their findings might validate the distinction between bipolar and unipolar types of affective disorder.

Singhal A K *et al*.[29] have investigated the role of stressful life events in mania, in 30 cases of acute mania. This work does show the relation of significant life stresses and family pathology in the genesis of mania. Significantly, death of a close relative/spouse, financial difficulties, disappointment of loss turned to be major life events.

Tapas K A *et al*.[30] have attempted to see the relation in Adolescent mania, EEG abnormalities and response to anti convulsant medication in a three-year follow-up study. Significant finding is - a large per cent (43.75%) had moderate to severe EEG abnormalities. Patients who continued anti convulsive mood stabilizers had relapsed very less compared to those who discontinued. This study highlights the value of prophylaxis especially in those adolescents having EEG abnormalities.

### Disability

H Taroor *et al*.[31] attempted to study the stability and quality of life in euthymic patients of bipolar affective disorder or recurrent depressive disorder with or without comorbid medical illnesses. This report shows the presence of chronic comorbid medical illness did not cause a difference in quality of life between the two groups during euthymia.

### Comorbidity

A few case reports of comorbid, other psychiatric illnesses in bipolar mood disorders were reported in Indian literature. Dysmorphophobia as a comorbid disorder occurring in both depressive episodes and manic episodes in a case of bipolar mood disorder was described by Sengupta *et al*.[32] The difficulty associated with both diagnosis and management was discussed. H Kalra *et al*.[33] have discussed a case of bipolar affective disorder with obsessive compulsive disorder as a comorbidity in manic phase of the disorder, the difficulties in the management was discussed.

### Treatment: Biological

There has been a large volume of research reported in the treatment aspects. There have been models to explain the treatment approaches and various clinical trials to show early evidence for various drugs both from experience and experimental.

Interestingly, the first report on treatment of manic depressive psychosis treated with long term electro convulsive therapy was by Bhaskaran,[34] who described usage of long term benefits of Electro convulsive treatment in a case of manic depressive psychosis and Venkoba Rao[35] also described a case of rapid cycling.

Lithium kinetics was studied by Pradhan N *et al*.[36] They have studied the differential pharmaco kinetics in 16 patients of manic depressive psychosis between serum and erythrocytes and explained based on their observations that there is a bi compartmental ‘model’ of plasma and erythrocyte reaching steady state and undergoing fluctuations and may exhibit more different half lines over a time. Essentially, a model of lithium kinetics was attempted to be explained.

N Desai *et al*.[37] Gangadhar B N *et al*.[38] have, in case reports, demonstrated the benefits of lithium and carbamazepine in a treatment resistant manic depressive psychosis. Prakash H M and S Bharath[39] in a first controlled double blind study have shown the efficacy of valproate in acute mania. In this study, lithium was compared to valproate in acute mania and both showed to equal efficacy in controlling mania symptoms. This study is possibly the first paper for evidence of efficacy.

I. P. Khalkho and Khess C. R. J[40] have attempted to identify the various factors of drug non compliance in mania patients, demographic clinical variables and personality variables using 16 personality factors questionnaire. Not so surprisingly, they found that commonest factor for non compliance was side effects of medicines followed by the feeling of ‘well being’. Pretension, jealousy, suspiciousness are the personality factors responsible for poor drug compliance leading the authors to conclude that patients with non compliance use less of mature defenses and more of primitive defenses.

### Recent evidence

The role of quetiapine monotherapy was presented in a case report by Khazaal Y[41] arguing for the building up evidence in double blind placebo control long term mood stabilizing studies.

Solanki R K *et al*.[42] have, in a one-week open label trial, shown the benefits of injectable sodium valproate in patients with mania and concluded that substantial improvement was seen and no major side effects were noticed. In this short study, utility in the acute stage was of mania of using injectable sodium valproate was argued for.

M Trivedi *et al*.[43] have, in a single case report, discussed the utility of resperidone mono therapy in the prophylaxis of bipolar affective disorder, further highlighting the need for double blind randomized placebo control studies.

Pradeep R J[44] has highlighted, in three case reports, the different types of delirium encountered in valproate induced hyper ammonemia states and management issues. In one of the cases, Pradeep has rechallenged valpraote-caused hyperammonia state validating the risk. The author highlights the clinical significance of watching for hyperammonia state as a cause of delirium where valproate was used.

In an interesting case report, V Agarwal and Tripathi[45] report of the utility of memantine as co pharmacy helped in a case of better tolerability and efficacy.

R Balon[46] has in his psychiatric pearls contribution has argued on the evidence that lithium is a unique mood stabilizer and meets the rigorous standards for mood stabilization in various forms of the bipolar mood disorder.

### Pharmacological evidence in special population

Khandelwal S K *et al*.[47] have reported prophylaxis benefits of lithium carbonate in children diagnosed as manic depressive psychosis. The work details in an open label study the utility, efficaciousness and tolerability of lithium carbonate in children diagnosed to have manic depressive psychosis and conclude that side effects are less and if serum levels of lithium are maintained between 0.6 and 1.2 m EQ/L.

Mohandas E and Rajmohan V[48] have in a recent C.M.E. topic reviewed the use of Lithium in special populations in detail of various medical disorders, pregnancy and lactation, in elderly and child and adolescents.

## IN SUMMARY

There has been a lot of research on bipolar mood disorders. Significantly, not many studies have been reported on biological, neuro imaging and genetic studies and long term course of bipolar disorders. There is also less replication of significant aspects of the bipolar mood disorder. Most studies have a small number as sample, the later studies methodologically improved. Multi centric studies done across the study with sound methodology and in various above areas of different areas will have to be done to generate the Indian data. Likewise, pharmacologically, the often repeated subjective to experience of optional dose of various medications will also have to be studied.

Transcultural differences have to be highlighted by attempting to do research in these areas. More naturalistic studies done across rural and urban background will help us to understand the course of bipolar in Indian context. The specific factors of psycho social and compliance issues in drug and therapy in the Indian context need to be studied.
